# Enhanced Recovery Pathway and Postoperative Ileus After Elective Minimally Invasive Colorectal Surgery

**DOI:** 10.3390/jcm15103895

**Published:** 2026-05-19

**Authors:** Codruta Craciun, Jenel Marian Patrascu, Danut Dejeu, Ana-Maria Davidoiu-Salavastru, Adrian Cosmin Ilie, Patricia Octavia Mazilu, Lavinia Craciun, Stelian Pantea

**Affiliations:** 1 Doctoral School, “Victor Babes” University of Medicine and Pharmacy Timisoara, 300041 Timisoara, Romania; codruta.craciun@umft.ro; 2 Orthopaedics II Research Center, “Pius Brinzeu” Emergency County Hospital, 300723 Timisoara, Romania; jenel.patrascu@umft.ro; 3 Department of Orthopedics and Traumatology, “Victor Babes” University of Medicine and Pharmacy, Efimie Murgu Square 2, 300041 Timisoara, Romania; 4 Surgical Oncology Department, Emergency County Hospital Oradea, 410169 Oradea, Romania; 5 Department of Functional Sciences, Discipline of Public Health, Center for Translational Research and Systems Medicine, “Victor Babes” University of Medicine and Pharmacy Timisoara, 300041 Timisoara, Romania; ilie.adrian@umft.ro; 6 Faculty of Medicine, “Victor Babes” University of Medicine and Pharmacy Timisoara, 300041 Timisoara, Romania; patrimazilu@yahoo.com; 7 Department of Anatomy and Embryology, “Victor Babes” University of Medicine and Pharmacy Timisoara, 300041 Timisoara, Romania; craciun.lavinia@umft.ro; 8 Department X, Surgical Emergencies Clinic, “Victor Babes” University of Medicine and Pharmacy Timisoara, 300041 Timisoara, Romania; pantea.stelian@umft.ro

**Keywords:** colorectal surgery, enhanced recovery after surgery, postoperative ileus, length of stay, opioid analgesics

## Abstract

**Background:** Postoperative ileus (POI) remains a leading driver of delayed recovery and prolonged length of stay (LOS) after colorectal surgery. Although ERAS is well established, less is known about how pathway adherence and implementation fidelity relate to bowel recovery in pragmatic minimally invasive practice. **Objectives:** To evaluate whether a structured ERAS pathway, delivered in routine care, was associated with lower POI and improved early recovery compared with contemporaneous standard care after elective minimally invasive colorectal surgery. **Methods:** In a prospective, non-randomized pragmatic comparative study conducted from January 2022 to September 2024, 123 adults undergoing elective laparoscopic colorectal resection were managed with either an ERAS pathway (*n* = 62) or standard care (*n* = 61). POI was operationalized prospectively using predefined clinical criteria and daily team assessment. Primary outcome was POI. Secondary outcomes included time to flatus, LOS, 48 h opioid use (morphine milligram equivalents, MME), complications (Clavien–Dindo), 30-day readmission, and Quality of Recovery (QoR-15). Multivariable logistic regression and propensity score–adjusted sensitivity analyses were performed to address baseline imbalance. **Results:** POI occurred in 7/62 (11.3%) in ERAS vs. 22/61 (36.1%) in standard care (*p* = 0.002). ERAS patients had earlier flatus (38.6 ± 15.2 h vs. 60.0 ± 20.1 h, *p* < 0.001), shorter LOS (4.2 [3.4–5.0] vs. 5.4 [4.5–6.8] days, *p* < 0.001), lower 48 h opioids (35.4 [25.2–47.8] vs. 61.1 [41.5–88.6] MME, *p* < 0.001), and higher QoR-15 at POD2 (113.9 ± 14.9 vs. 104.8 ± 15.5, *p* = 0.001). In the primary multivariable model, ERAS was independently associated with lower POI odds (adjusted OR 0.2; 95% CI 0.1–0.7; *p* = 0.013); the association remained directionally similar in propensity-adjusted sensitivity analysis (adjusted OR 0.31; 95% CI 0.12–0.79; *p* = 0.015). Higher adherence was associated with lower POI and lower opioid exposure. **Conclusions:** In this prospective cohort, ERAS implementation was associated with lower POI incidence and faster early recovery; however, findings should be interpreted as observational and hypothesis-generating rather than causal.

## 1. Introduction

Postoperative ileus (POI), a transient impairment of coordinated gastrointestinal motility after surgery, remains one of the most frequent and clinically consequential barriers to timely recovery after colorectal resection. Contemporary reviews highlight that POI (and prolonged POI in particular) is common after colorectal surgery and is strongly associated with delayed tolerance of oral intake, nausea/vomiting, impaired mobilization, prolonged length of stay (LOS), and greater risk of downstream complications linked to immobility and supportive-care escalation (intravenous fluids, nasogastric decompression) [1]. Mechanistically, POI is increasingly understood as a multifactorial, phase-dependent stress response rather than a single-pathway phenomenon. Experimental and clinical synthesis work supports an interplay between early neurogenic inhibition and later inflammatory and immune-mediated suppression of smooth-muscle activity, with additional contributions from surgical handling, sympathetic activation, and exogenous drugs that impair enteric signaling [2]. This biologic redundancy helps explain why isolated interventions often yield variable effects and why bundled strategies that target multiple contributors simultaneously are conceptually attractive for POI prevention and mitigation [2].

Beyond biology, prolonged POI reflects a convergence of patient-, procedure-, and care-process factors. In colorectal cancer surgery cohorts, multivariable analyses have identified associations between prolonged POI and perioperative exposures that plausibly intensify inflammatory load or suppress motility, reinforcing that “gut recovery” is sensitive to both operative stress and modifiable perioperative management choices [3]. These data support a pragmatic focus on care pathways that standardize best practices across the full perioperative timeline, rather than relying on ad hoc, provider-dependent decisions [3]. The health-system burden of prolonged POI is substantial. Prospective economic analyses using standardized diagnostic definitions demonstrate that prolonged POI is associated with significantly increased inpatient costs across multiple domains (e.g., ward care, imaging, medications, laboratory testing, allied health services), and remains an independent financial burden even when accounting for major complications and LOS [4]. Because value-based surgical care increasingly targets potentially preventable drivers of prolonged hospitalization, POI has become a high-priority endpoint for perioperative quality improvement initiatives [4].

Enhanced Recovery After Surgery (ERAS) programs are standardized, multimodal pathways intended to reduce surgical stress, preserve physiologic function, and accelerate return to baseline activity. The ERAS Society’s colorectal surgery recommendations (2018 update) synthesize graded evidence supporting interventions such as structured patient education, avoidance of routine tubes/drains, goal-directed fluid therapy, opioid-sparing multimodal analgesia, early feeding, and early mobilization, elements that align directly with known contributors to POI [5]. Implementation fidelity is therefore central to the real-world effectiveness of ERAS. Health-system implementation studies describe ERAS as an integrated model combining evidence-based guidelines, structured change management, and continuous audit/feedback to support sustained practice transformation across institutions and teams [6]. This perspective is particularly relevant in minimally invasive colorectal surgery, where baseline recovery is already optimized, and the incremental benefit of ERAS may depend on achieving a sufficient “dose” of pathway adherence to meaningfully reduce opioids, accelerate mobilization, and normalize feeding and bowel function [6].

Evidence syntheses and comparative trials support the clinically meaningful benefits of enhanced recovery strategies in colorectal surgery, including reductions in overall morbidity and shorter LOS without increased readmissions in meta-analyses of randomized trials [7]. Randomized data further suggest that combining laparoscopy with fast-track multimodal management can outperform either strategy alone, highlighting potential synergy between minimally invasive operative approaches and pathway-driven perioperative care [8]. Nonetheless, outcome variability across settings persists, emphasizing the need to evaluate ERAS effects within specific institutional contexts and implementation models [7,8].

At the institutional level, detailed pathway descriptions underscore that operationalizing ERAS involves aligning anesthetic, surgical, and postoperative practices into a coherent workflow with clear ownership and compliance monitoring [9]. International audit infrastructure has also matured, with large-scale web-based databases enabling benchmarking and supporting the principle that higher overall compliance correlates with better outcomes and lower costs of care across participating centers [10].

Opioid exposure remains a key mediator linking perioperative processes to bowel recovery. Contemporary ERAS-focused analgesia reviews emphasize that opioids can delay return of bowel function and contribute to nausea/vomiting and sedation, while multimodal, opioid-sparing regimens (systemic non-opioids and regional/neuraxial techniques) can reduce opioid requirements and may lessen ileus risk [11]. Supporting evidence includes meta-analytic comparisons indicating that epidural analgesia can improve analgesia and is associated with reduced ileus versus parenteral opioid-based strategies, albeit with trade-offs and heterogeneous effects on LOS [12]. Pharmacologic strategies also exist, most notably the peripherally acting μ-opioid receptor antagonist alvimopan, which, in pooled phase III analyses, has been shown to accelerate gastrointestinal recovery and reduce consequences of POI after bowel resection [13]. In practice, however, the overall recovery signal may depend on how such measures integrate with broader pathway elements such as early feeding, mobilization, and fluid stewardship [11,12,13]. Parallel to traditional clinical endpoints, patient-reported outcomes (PROs) provide complementary information on recovery quality that may not be captured by LOS alone. The Quality of Recovery-15 (QoR-15) was developed as a short, psychometrically robust instrument that correlates with surgical stress and LOS while remaining feasible for routine clinical use [14]. Subsequent work supports its acceptability and responsiveness in perioperative settings, reinforcing its utility for capturing patient-centered recovery trajectories alongside physiologic milestones such as return of bowel function [15].

Accordingly, the present study aimed to examine, in a pragmatic real-world setting, whether a structured ERAS pathway and the degree of pathway adherence were associated with POI, opioid exposure, and early patient-centered recovery after elective minimally invasive colorectal surgery, compared with contemporaneous standard care.

## 2. Materials and Methods

### 2.1. Study Design and Participants

This prospective comparative study was conducted at a tertiary-care surgical center with an established minimally invasive colorectal program between January 2022 and September 2024. During this interval, the service performed approximately 140–160 minimally invasive colorectal resections annually; the present analysis included consecutive elective laparoscopic cases meeting prespecified eligibility criteria and complete 30-day follow-up. The design was pragmatic and implementation-focused: patients were treated within routine care pathways rather than randomized, with the aim of evaluating real-world pathway delivery and adherence. The authors used ChatGPT v4.0, an AI language model developed by OpenAI (San Francisco, CA, USA), to exclusively improve the manuscript’s language and readability. All the scientific content, interpretations, and conclusions are the original work of the authors.

Eligibility criteria included age ≥ 18 years, elective minimally invasive colorectal resection (right colectomy, left/sigmoid colectomy, or anterior resection), and the ability to provide informed consent and complete PRO assessments. Exclusion criteria included emergency surgery, planned open surgery at the outset, concurrent major non-colorectal procedures, preoperative bowel obstruction requiring decompression, chronic intestinal pseudo-obstruction, and inability to complete follow-up through 30 days.

A total of 123 patients were included: 62 managed with the ERAS pathway and 61 with standard care. Baseline assessment included demographics, comorbidity burden (Charlson comorbidity index), American Society of Anesthesiologists (ASA) class, smoking status, diabetes status, and preoperative laboratory values (hemoglobin and albumin). Procedure type and intraoperative variables were recorded prospectively.

### 2.2. Perioperative Care Pathways

The ERAS pathway consisted of coordinated preoperative education, goal-directed intraoperative fluid management, regional analgesia when feasible (including transversus abdominis plane (TAP) block), avoidance of routine nasogastric tube placement, and an opioid-sparing multimodal analgesia regimen. Postoperatively, the pathway emphasized early oral intake (by postoperative day (POD) 1), early mobilization (ambulation by POD1), early urinary catheter removal when clinically appropriate, and standardized nausea/vomiting prophylaxis. These components were selected to target recognized contributors to delayed gut motility and immobility.

Standard care reflected the contemporaneous institutional baseline pathway before formal ERAS scale-up. Usual-care elements included postoperative diet advancement at the clinician’s discretion (typically after bowel sounds or flatus), mobilization encouraged but not protocol-timed, urinary catheter removal usually after POD1–2, depending on surgeon preference, selective nasogastric tube use, and analgesia more frequently centered on systemic opioids without a mandatory regional block component. These usual-care components were documented prospectively to improve reproducibility of the comparator, but standard care did not include formal audit-and-feedback, a checklist-based workflow, or predefined adherence targets. Accordingly, the comparison should be interpreted as structured pathway delivery versus contemporaneous non-protocolized routine care, with the possibility of performance bias acknowledged explicitly.

Implementation fidelity was captured using an ERAS adherence score (0–10), reflecting completion of key pathway elements. In addition, binary indicators were recorded for core components: early feeding, early mobilization, avoidance of nasogastric tubes, early Foley catheter removal, and prophylactic antiemetics. These process measures were used to characterize the pathway “dose” and contextualize outcome differences.

### 2.3. Outcomes and Definitions

The primary endpoint was postoperative ileus (POI). To reduce subjectivity, POI was operationalized prospectively as a deviation from the expected postoperative course on or after POD4 requiring a change in management and accompanied by at least two of the following: persistent intolerance of oral intake, nausea or vomiting, abdominal distension, absence of flatus/stool progression, or need for nasogastric decompression, after exclusion of mechanical obstruction, anastomotic leak, or intra-abdominal sepsis. Daily POI assessment was performed by the treating surgical team using a standardized ward-round case-report form completed by the resident and validated by the attending surgeon. Because pathway allocation was evident to clinicians, blinding of bedside assessors was not feasible. Borderline cases were reviewed the same day by two senior colorectal surgeons not involved in the index operation, and final classification was reached by consensus. This operational definition was prospectively aligned with the symptom domains emphasized in the Vather criteria and with later postoperative ileus core-outcome standardization efforts.

Secondary outcomes included length of stay (LOS, days), 0–48 h opioid consumption expressed as morphine milligram equivalents (MME), POD1 pain score (0–10), postoperative nausea and vomiting (PONV), surgical site infection (SSI), anastomotic leak, overall complications (Clavien–Dindo ≥ II), major complications (Clavien–Dindo ≥ III), and 30-day readmission. Patient-reported recovery was measured using the QoR-15 at POD2 and day 7, with higher scores indicating better recovery.

To support subgroup interpretation, outcomes were also examined within procedure categories (right colectomy, left/sigmoid colectomy, anterior resection). Because procedure types differ in operative extent and pelvic dissection, subgroup analyses were considered clinically relevant and were reported with interaction testing to evaluate heterogeneity in the ERAS association.

### 2.4. Statistical Analysis

Continuous variables were summarized as mean ± standard deviation (SD) when approximately symmetric and as median [interquartile range (IQR)] when skewed. Categorical variables were summarized as *n* (%). Between-group comparisons used Welch’s *t*-test for continuous approximately normal variables, and Mann–Whitney U tests for skewed variables (e.g., LOS, blood loss, opioid use). Categorical variables were compared using χ^2^ tests; Fisher’s exact test was used when expected cell counts were small.

For subgroup analyses by procedure type, within-procedure comparisons of ERAS vs. standard care used Fisher’s exact test (POI) and Mann–Whitney U tests (LOS and opioid use). Interaction was assessed using likelihood-ratio testing for POI (logistic regression models with and without the group-by-procedure interaction) and nested model comparison on log-transformed LOS (linear regression models with and without interaction).

Correlations among key recovery and process variables were assessed using Spearman’s rank correlation coefficient (ρ), with significance annotated as * *p* < 0.05, ** *p* < 0.01, and *** *p* < 0.001. Multivariable logistic regression was used to identify independent associations with POI. Predictors were selected a priori based on clinical plausibility and included ERAS exposure, age, ASA class, operative time, conversion, and opioid exposure. Model discrimination was summarized using the area under the receiver operating characteristic curve (AUC). Statistical significance was defined as two-sided *p* < 0.05.

Given the non-randomized design and the baseline differences in the Charlson comorbidity index and albumin, additional sensitivity analyses were prespecified. A propensity score for ERAS exposure was estimated using age, Charlson index, albumin, ASA class, procedure type, and cancer indication; this score was entered as an adjustment covariate in a sensitivity logistic model for POI, with confirmatory inverse-probability weighting. Additional exploratory analyses examined ERAS adherence as a continuous predictor and by adherence quartiles to assess dose–response relationships. Attenuation of the adherence estimate after adjustment for 0–48 h opioid exposure was interpreted only as exploratory evidence compatible with mediation, not as a formal causal mediation analysis.

Because only 29 POI events occurred, the primary multivariable model was intentionally parsimonious to reduce overfitting. Secondary endpoints and subgroup comparisons were considered exploratory, and no formal multiplicity correction was applied; these results were therefore interpreted cautiously and with emphasis on effect size, consistency, and clinical plausibility rather than on isolated *p*-values.

## 3. Results

### 3.1. Patient Characteristics

Baseline characteristics are shown in Table 1. The cohort included 123 patients undergoing elective minimally invasive colorectal resections, with similar distributions of sex, BMI, ASA class, smoking status, cancer indication, and procedure types between groups. The standard-care group had a higher comorbidity burden and slightly lower albumin levels, which were considered in adjusted analyses.

Table 1 summarizes baseline case-mix and demonstrates that the two groups were broadly comparable in demographic and procedural composition, which is essential for interpreting pathway-associated differences in recovery. Age, sex distribution, and BMI were similar, with only a modest, non-significant trend toward older age in the standard-care group (65.4 ± 12.0 vs. 61.8 ± 10.0 years; *p* = 0.075). Procedural mix (right colectomy, left/sigmoid colectomy, and anterior resection) did not differ significantly overall (*p* = 0.087), although right colectomy was numerically more common in the ERAS arm. The most notable baseline imbalance involved comorbidity burden: the Charlson comorbidity index was higher in standard care (median 2.0 [1.0–3.0]) than ERAS (median 1.0 [1.0–2.0]; *p* < 0.001), suggesting that standard-care patients carried greater chronic disease burden that could predispose to slower recovery and complications independent of pathway effects. Preoperative albumin was also lower in standard care (3.8 ± 0.5 vs. 4.0 ± 0.4 g/dL; *p* = 0.012), consistent with slightly reduced physiologic reserve. In contrast, other risk factors with potential relevance to bowel recovery—such as ASA III status, smoking, diabetes, and cancer indication—were balanced, reducing concern for systematic confounding from these variables. Taken together, Table 1 indicates that observed outcome differences should be interpreted in the context of modest baseline risk enrichment in the standard-care group, motivating the inclusion of clinically important covariates in multivariable models and emphasizing the pragmatic, real-world nature of this comparative study.

Although overall procedure distribution did not differ statistically, the ERAS group contained a larger proportion of right colectomy and the standard-care group a larger proportion of left/sigmoid colectomy. Because procedure type may influence bowel recovery, subgroup and interaction analyses were prespecified, and baseline imbalances were additionally addressed in sensitivity models rather than ignored on the basis of *p*-values alone.

### 3.2. Perioperative Care Delivery and Pathway Implementation

Table 2 describes expected between-group differences in perioperative care delivery that define pathway implementation rather than independent efficacy endpoints.

Table 2 characterizes the operative context and, critically, quantifies the “dose” of pathway implementation through adherence metrics and discrete process elements. Operative time was slightly shorter in ERAS (157.7 ± 41.9 min) compared with standard care (172.7 ± 50.9 min), but this difference did not reach statistical significance (*p* = 0.077), suggesting that operative efficiency alone is unlikely to explain downstream recovery differences. Estimated blood loss and conversion rates were comparable, supporting similar technical complexity across groups. A prominent intraoperative distinction was fluid administration: ERAS patients received significantly less IV fluid (1561.7 ± 475.5 vs. 1850.5 ± 583.9 mL; *p* = 0.003), consistent with goal-directed strategies intended to reduce bowel edema and facilitate earlier gut function. Process adherence differences were marked and internally coherent. TAP block utilization—a core opioid-sparing component—was substantially higher under ERAS (72.6% vs. 16.4%; *p* < 0.001), indicating meaningful analgesic practice change rather than nominal pathway labeling. The ERAS adherence score reinforced this, with a large between-group separation (8.1 ± 1.3 vs. 4.3 ± 1.2; *p* < 0.001), suggesting bundled implementation rather than isolated changes. Postoperative behavior targets central to POI prevention also differed: early oral intake, early mobilization, avoidance of nasogastric tubes, and early Foley removal were all more common in ERAS (all *p* < 0.001). Notably, PONV prophylaxis did not differ significantly (*p* = 0.369), implying that the principal behavioral divergence involved feeding, mobility, tube/catheter management, fluids, and opioid-sparing analgesia. Overall, Table 2 demonstrates that the ERAS group experienced a distinctly different perioperative environment that is mechanistically aligned with improved gastrointestinal recovery and provides a credible process foundation for interpreting clinical outcome differences.

These process measures are therefore interpreted descriptively as markers of implementation fidelity and pathway delivery, not as stand-alone clinical outcomes attributable to ERAS.

### 3.3. Postoperative Recovery and Clinical Outcomes

Postoperative outcomes are summarized in Table 3. As exploratory between-group comparisons in a non-randomized cohort, these findings should be interpreted as associations observed within the study’s implementation context.

Table 3 provides the core clinical evidence linking ERAS implementation to measurable recovery benefits after abdominal surgery. Gastrointestinal recovery endpoints showed the most pronounced separation. ERAS patients achieved first flatus approximately 21 h earlier (38.6 ± 15.2 vs. 60.0 ± 20.1 h; *p* < 0.001), supporting a clinically meaningful acceleration of motility recovery rather than a marginal change. This translated into shorter LOS, with a median reduction of 1.2 days (4.2 [3.4–5.0] vs. 5.4 [4.5–6.8]; *p* < 0.001), a difference that is operationally significant for bed utilization and patient experience. Opioid exposure—an actionable mediator—was substantially lower in ERAS (35.4 [25.2–47.8] vs. 61.1 [41.5–88.6] MME; *p* < 0.001), while POD1 pain scores were similar (*p* = 0.141), suggesting that opioid reduction did not come at the cost of inferior early pain control. The primary endpoint, POI, occurred far less often under ERAS (11.3% vs. 36.1%; *p* = 0.002), representing an absolute risk reduction that is clinically compelling and consistent with the pathway’s mechanistic targets (fluids, opioids, mobility, early feeding). Importantly, broader morbidity followed the same pattern: any complication (Clavien–Dindo ≥II) was reduced (19.4% vs. 44.3%; *p* = 0.003), and major complications trended lower (3.2% vs. 13.1%; *p* = 0.054). SSI and leak rates were similar, implying that the ERAS benefit was driven more by functional recovery and non-infectious morbidity than by differences in anastomotic integrity or wound infection. Patient-reported recovery also improved substantially, with higher QoR-15 scores at POD2 and day 7, indicating that the ERAS-associated gains were not limited to administrative metrics but reflected a more favorable patient-perceived recovery trajectory.

### 3.4. Subgroup Analyses by Procedure Type

Procedure-stratified outcomes are shown in Table 4. ERAS-associated differences were directionally consistent across procedure types, with the largest POI reduction observed in left/sigmoid colectomy. Formal interaction testing did not demonstrate statistically significant heterogeneity.

Table 4 explores whether the association between ERAS and recovery differs by the type of abdominal procedure, a clinically meaningful question because the extent of bowel handling, anastomotic location, and pelvic dissection can influence ileus risk. Across all three procedure categories, ERAS is consistently associated with lower opioid requirements, with statistically significant reductions in right colectomy and left/sigmoid colectomy, and a similar direction of effect in anterior resection. This consistency strengthens the interpretation that opioid-sparing analgesia and pathway execution are robust across operative subtypes rather than limited to a single procedure. The POI signal appears strongest in left/sigmoid colectomy (8.0% vs. 38.7%; *p* = 0.014), where pelvic manipulation is limited compared to anterior resection, yet bowel handling and mobilization may still be substantial; this pattern suggests that pathway-driven improvements in fluids, feeding, and mobilization may be particularly effective when baseline ileus risk is moderate and modifiable. For right colectomy and anterior resection, POI differences were directionally favorable but did not reach statistical significance, likely reflecting smaller subgroup sizes and reduced power rather than true absence of effect. LOS differences mirror the POI pattern, with significant shortening for right colectomy and left/sigmoid colectomy, and a smaller, non-significant difference in anterior resection. Importantly, interaction testing did not show statistically significant heterogeneity for either POI or LOS, indicating that, within the limits of sample size, ERAS benefits are broadly applicable across procedure types rather than restricted to one. In practice, these findings support pathway deployment as a default strategy across minimally invasive colorectal resections, while also highlighting that effect visibility depends on subgroup sample size and baseline risk, which can obscure clinically relevant trends in smaller procedure strata.

### 3.5. Correlations Among Pathway Dose and Recovery Metrics

Spearman correlations between pathway/process measures and recovery outcomes are shown in Table 5.

Table 5 links pathway “dose” and mechanistic mediators to downstream recovery outcomes using rank-based correlations that are robust to non-normal distributions. Higher ERAS adherence score correlates moderately with lower opioid exposure (ρ = −0.5; *p* < 0.001), shorter flatus time (ρ = −0.4; *p* < 0.001), and shorter LOS (ρ = −0.5; *p* < 0.001). These relationships reinforce that the pathway is not merely a label but a measurable behavioral package whose degree of completion aligns with clinically meaningful recovery improvements. ERAS adherence is also positively correlated with better early patient-reported recovery (QoR-15 at POD2; ρ = 0.3; *p* < 0.001), suggesting that the physiologic benefits of faster gut recovery and lower opioid use are reflected in patient-perceived well-being. Opioid exposure shows a clinically coherent pattern: it correlates with delayed gastrointestinal recovery (ρ = 0.2; *p* < 0.05 for flatus time) and with longer LOS (ρ = 0.4; *p* < 0.001). Moreover, higher opioids associate with worse QoR-15 at POD2 (ρ = −0.2; *p* < 0.05), indicating that opioid minimization may improve both objective and subjective recovery endpoints. Flatus time and LOS correlate strongly (ρ = 0.4; *p* < 0.001), supporting the conceptual model that delayed motility appears as a direct operational driver of discharge readiness. Comorbidity burden (Charlson index) demonstrates a smaller but significant positive association with LOS (ρ = 0.2; *p* < 0.05), consistent with reduced physiologic reserve and slower recovery among higher-risk patients. Operative time correlates weakly with LOS (ρ = 0.2; *p* < 0.05) and inversely with QoR-15 (ρ = −0.2; *p* < 0.05), suggesting that longer operations may modestly worsen early subjective recovery and extend hospitalization. Collectively, Table 5 supports a plausible causal chain—higher ERAS adherence → lower opioids → faster gut recovery → shorter LOS and better QoR—while also indicating that patient complexity and operative duration contribute incremental variance.

### 3.6. Multivariable and Sensitivity Analyses of Postoperative Ileus

Multivariable logistic regression results for POI are shown in Table 6. The primary model was intentionally parsimonious because of the limited number of POI events. Sensitivity analyses addressing baseline imbalance and adherence-response patterns are reported below.

Table 6 evaluates independent associations with POI after accounting for patient and operative factors, providing inferential support beyond unadjusted comparisons. The ERAS pathway remains significantly associated with reduced POI risk (adjusted OR 0.2; 95% CI 0.1–0.7; *p* = 0.013), indicating that the observed reduction in POI is not fully explained by baseline differences in age, ASA class, operative time, or conversion. This finding is clinically meaningful: an odds ratio near 0.2 corresponds to a large relative reduction in the likelihood of ileus and aligns with the mechanistic intent of ERAS to preserve gut function through opioid-sparing analgesia, early mobilization, fluid optimization, and early feeding. Opioid exposure emerges as an independent risk marker (adjusted OR 1.7 per 25 MME; 95% CI 1.2–2.5; *p* = 0.006), reinforcing the biological plausibility that higher opioid dosing contributes to impaired gut motility and delayed bowel function. The magnitude of this association is actionable, suggesting that relatively modest increments in early postoperative opioid dosing may meaningfully shift ileus risk. Other covariates (age, ASA III, operative time, conversion) were not statistically significant in this model, which may reflect limited event counts, collinearity with pathway-related processes, or a true dominance of opioid/pathway effects in this cohort. The model’s discrimination (AUC = 0.8) is acceptable for clinical prediction in a modest sample and supports that the included predictors capture relevant variance in POI occurrence.

### 3.7. Adherence–Outcome Gradient

Sensitivity analyses addressing baseline imbalance supported the main findings. In a propensity score–adjusted model for POI that included age, Charlson index, albumin, ASA III, procedure type, and cancer indication, ERAS remained associated with lower POI odds (adjusted OR 0.31; 95% CI 0.12–0.79; *p* = 0.015). Results were similar in inverse-probability weighting (adjusted OR 0.34; 95% CI 0.14–0.81; *p* = 0.016). When adherence was modeled continuously, each 1-point increase in adherence score was associated with lower odds of POI (OR 0.68; 95% CI 0.52–0.90; *p* = 0.007); after additional adjustment for 0–48 h opioid exposure, this estimate attenuated (OR 0.81; 95% CI 0.60–1.08; *p* = 0.148), consistent with a plausible but exploratory opioid-mediated pathway. Because of the limited event count, these analyses are hypothesis-generating and should not be interpreted as proof of mediation (Table 7).

A graded pattern was observed, with higher adherence associated with lower POI incidence, lower opioid exposure, shorter LOS, and better QoR-15. Because adherence and opioid exposure were strongly correlated, mediation findings should be interpreted as exploratory rather than definitive.

**Figure 1:**
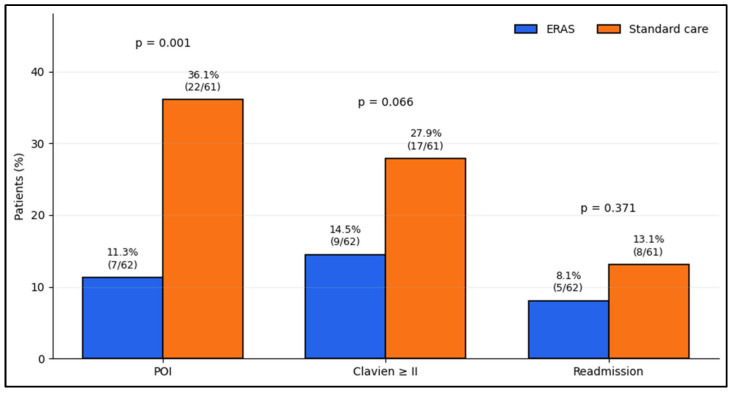
Postoperative outcomes by care pathway.

Figure 1 illustrates the between-group distribution of selected postoperative outcomes. POI was less frequent in ERAS than in standard care (11.3% [7/62] vs. 36.1% [22/61], *p* = 0.002). Differences in Clavien–Dindo ≥ II complications and 30-day readmission should be interpreted cautiously, given the observational design and limited event counts.

**Figure 2:**
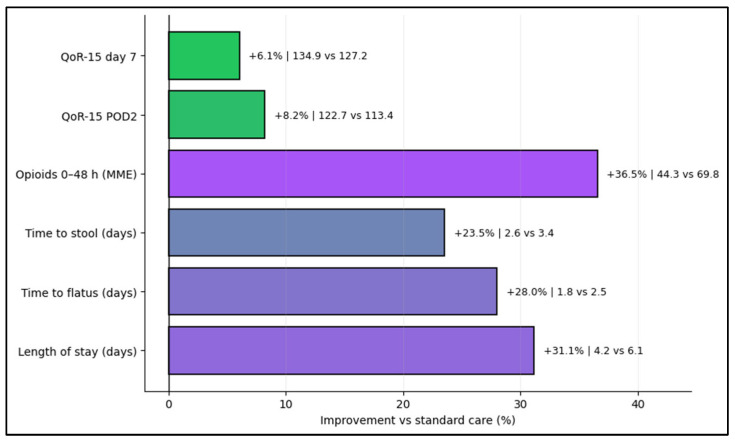
Recovery advantages with ERAS vs. standard care.

Figure 2 summarizes relative differences in recovery metrics between pathways. The largest separations were observed for opioid exposure at 0–48 h and length of stay, with concordant differences in time to flatus and QoR-15. These graphical summaries are intended to illustrate the direction and coherence of associations rather than establish causality.

## 4. Discussion

This study should be interpreted primarily as a pragmatic implementation analysis rather than a de novo efficacy trial. The overall direction of findings—lower POI, earlier flatus, lower opioid exposure, shorter LOS, and better early QoR-15 under ERAS—is concordant with randomized and meta-analytic colorectal ERAS literature [7,8,15,16,17,18,19]. The more distinctive contribution of the present cohort is that these associations were observed within a routine minimally invasive colorectal program and were accompanied by a clear adherence gradient. In other words, the study adds less by asking whether ERAS can work in principle, and more by showing that how reliably ERAS is delivered in daily practice may be closely linked to whether clinically meaningful bowel-recovery differences are actually seen. This interpretation is consistent with implementation reports and large audit experiences showing that compliance, feedback, and pathway ownership are central to measurable ERAS benefit [20,21,22,23].

The magnitude of the observed POI difference nonetheless warrants careful interpretation. Compared with some published colorectal cohorts, the absolute reduction appears relatively large; however, POI incidence varies substantially according to the definition applied, the timing of assessment, and whether the endpoint captures any clinically relevant deviation in gut recovery or only more prolonged and severe dysfunction [16,24,25,26,27,28,29,30,31]. That methodological variability is one reason why cross-study comparisons are difficult and why reviewers’ concerns about soft endpoints are justified. In the revised manuscript, we therefore made the bedside definition more explicit, mapped it to the symptom domains emphasized by Vather and colleagues, and referenced the subsequent core outcome standardization effort [31,32]. Even with this clarification, some detection and classification bias remains possible because POI was assessed prospectively but without assessor blinding. The fact that the POI signal was accompanied by concordant differences in flatus time, opioid use, LOS, and QoR-15 strengthens clinical coherence, but it does not eliminate the possibility that part of the apparent effect size reflects how the endpoint was operationalized.

The comparator structure also matters. Several variables shown in Table 2—early feeding, mobilization, catheter removal, tube avoidance, fluid stewardship, and opioid-sparing analgesia—are intrinsic components of the ERAS bundle and should therefore be read as fidelity descriptors rather than independent efficacy outcomes. Reviewer 3 correctly highlighted that presenting these items as if they were separate findings could overstate inference. The more appropriate interpretation is that the study contrasts a structured, audited, checklist-driven pathway with explicit adherence targets against contemporaneous clinician-directed routine care without formal audit-and-feedback [6,9]. That distinction has two implications. First, the observed differences may reflect both the content of ERAS and the discipline of standardized implementation. Second, the variability of standard care limits reproducibility and introduces performance bias, which we now acknowledge explicitly in both the Methods and the Limitations. Similar lessons have been emphasized in the implementation of the science literature, showing that protocolization and monitoring are integral parts of ERAS effectiveness, not merely background context [21,22,23].

Residual confounding remains a central reason to avoid causal language. The standard-care group entered surgery with a higher comorbidity burden, lower albumin, and a somewhat different procedure mix, all of which may independently worsen recovery even when minimally invasive techniques are used. The sensitivity analyses were directionally reassuring, but propensity adjustment and weighting can only address measured covariates. Unmeasured frailty, disease severity, prehabilitation engagement, surgeon preference, and ward culture could still have influenced both pathway allocation and postoperative recovery. Procedure-specific analyses partly mitigate this concern by showing generally similar directions of association across right colectomy, left/sigmoid colectomy, and anterior resection, yet those subgroup comparisons were modest in size and not powered to exclude meaningful heterogeneity. This cautious reading aligns with prior literature showing that prolonged POI risk is shaped not only by perioperative care but also by patient complexity and operative subtype [3,20,27,28].

Opioid exposure appears to be one of the most plausible mechanistic links between pathway delivery and bowel recovery. In the present cohort, ERAS patients received substantially less opioid analgesia over the first 48 h while reporting similar POD1 pain scores, suggesting that bowel recovery was not improved simply by undertreating pain. This pattern is biologically credible and consistent with ERAS analgesia literature showing that opioids delay gastrointestinal recovery, whereas multimodal regimens and regional techniques may reduce ileus risk without compromising analgesia [11,12]. The exploratory attenuation of the adherence–POI association after adjustment for opioid exposure is likewise compatible with partial mediation, although the study was not powered for a formal mediation model, and the result should not be overinterpreted. TAP-block use was far higher in ERAS, and prior randomized and observational work supports TAP blocks as practical opioid-sparing adjuncts within laparoscopic colorectal pathways [24,25,26]. Collectively, these findings make analgesic strategy one of the most actionable modifiable elements of implementation [27].

Patient-reported recovery adds an important layer of interpretation. Length of stay can shorten for operational reasons alone, but the parallel improvement in QoR-15 at POD2 and day 7 suggests that the ERAS-associated recovery signal in this cohort was not merely administrative. Earlier flatus, lower opioid exposure, shorter admission, and better QoR-15 together support a broader recovery phenotype in which bowel function, comfort, mobility, and perceived well-being improve in parallel. This is consistent with the psychometric rationale of QoR-15 and with colorectal ERAS literature showing that patient-reported measures complement traditional surgical endpoints and help distinguish earlier discharge from genuinely better convalescence [14,15,30].

From a translational standpoint, the present results support framing ERAS less as a binary exposure and more as an implementation-quality construct. That perspective is especially relevant in minimally invasive colorectal surgery, where baseline outcomes are already relatively favorable, and the remaining opportunity may lie in reducing variation in pathway delivery rather than inventing entirely new perioperative components. Future research should therefore prioritize multicenter implementation studies, procedure-specific tailoring, and standardized bowel-recovery outcome frameworks, ideally incorporating the emerging core outcome set so that adherence, POI, complications, LOS, and patient-centered recovery can be compared more reliably across institutions [21,22,23,29,32]. Nevertheless, these findings should be interpreted in light of potential residual confounding from unmeasured or incompletely controlled factors, including underlying comorbidities and other patient- and treatment-related characteristics [33,34,35,36,37,38,39,40,41,42].

Several limitations remain important. The study was prospective but non-randomized, the comparator pathway was less standardized, and bedside POI assessment could not be blinded. Accordingly, selection bias, performance bias, and some misclassification of functional recovery endpoints remain possible despite prespecified criteria and consensus adjudication of borderline cases. The relatively large between-group differences in POI and overall complications should also be interpreted in light of the fact that hard surgical events, such as anastomotic leak and SSI, were similar, implying that the strongest observed benefits were concentrated in softer or recovery-related outcomes. In addition, multiple secondary and subgroup analyses were exploratory, no formal multiplicity adjustment was applied, and the limited number of POI events constrained model complexity. These issues do not negate the internal coherence of the overall signal, but they do support an association-based and hypothesis-generating interpretation rather than a causal one.

## 5. Conclusions

In this prospective pragmatic cohort of elective minimally invasive colorectal surgery, higher-fidelity ERAS delivery was associated with lower POI incidence, lower early opioid exposure, faster bowel recovery, shorter LOS, and better early QoR-15. The adherence–outcome gradient suggests that implementation quality and opioid-sparing care processes may be key modifiable targets. Because the comparator was non-randomized and less protocolized, these findings should be interpreted as hypothesis-generating associations that warrant confirmation in multicenter, standardized, and preferably randomized or stepped-wedge studies.

## Figures and Tables

**Table 1:** Baseline demographics and clinical characteristics.

Variable	ERAS (*n* = 62)	Standard (*n* = 61)	*p*-Value
Age, years	61.8 ± 10.0	65.4 ± 12.0	0.075
Female sex	30 (48.4)	25 (41.0)	0.394
BMI, kg/m^2^	28.2 ± 4.5	27.9 ± 5.0	0.708
ASA class III	23 (37.1)	27 (44.3)	0.411
Charlson comorbidity index	1.0 [1.0–2.0]	2.0 [1.0–3.0]	<0.001
Diabetes mellitus	13 (21.0)	18 (29.5)	0.277
Current smoker	11 (17.7)	12 (19.7)	0.847
Hemoglobin, g/dL	13.1 ± 1.3	12.6 ± 1.5	0.088
Albumin, g/dL	4.0 ± 0.4	3.8 ± 0.5	0.012
Cancer indication	42 (67.7)	41 (67.2)	0.952
Procedure type			0.087
• Right colectomy	24 (38.7)	15 (24.6)	
• Left/sigmoid colectomy	25 (40.3)	31 (50.8)	
• Anterior resection	13 (21.0)	15 (24.6)	

**Table 2:** Perioperative care delivery and pathway implementation characteristics.

Variable	ERAS (*n* = 62)	Standard (*n* = 61)	*p*-Value
Operative time, min	157.7 ± 41.9	172.7 ± 50.9	0.077
Estimated blood loss, mL	91.3 [52.9–130.5]	85.4 [52.5–158.3]	0.630
Conversion to open	2 (3.2)	4 (6.6)	0.435
Intraoperative IV fluids, mL	1561.7 ± 475.5	1850.5 ± 583.9	0.003
TAP block performed	45 (72.6)	10 (16.4)	<0.001
ERAS adherence score (0–10)	8.1 ± 1.3	4.3 ± 1.2	<0.001
Early oral intake (≤POD1)	46 (74.2)	16 (26.2)	<0.001
Early mobilization (≤POD1)	47 (75.8)	21 (34.4)	<0.001
No nasogastric tube	56 (90.3)	39 (63.9)	<0.001
Foley catheter out (≤POD1)	44 (71.0)	20 (32.8)	<0.001
PONV prophylaxis administered	49 (79.0)	44 (72.1)	0.369

**Table 3:** Postoperative recovery and 30-day outcomes.

Outcome	ERAS (*n* = 62)	Standard (*n* = 61)	*p*-Value
Time to first flatus, h	38.6 ± 15.2	60.0 ± 20.1	<0.001
Length of stay, days	4.2 [3.4–5.0]	5.4 [4.5–6.8]	<0.001
Opioid use 0–48 h, MME	35.4 [25.2–47.8]	61.1 [41.5–88.6]	<0.001
Pain score POD1 (0–10)	4.1 ± 1.6	4.5 ± 1.6	0.141
QoR-15 POD2 (0–150)	113.9 ± 14.9	104.8 ± 15.5	0.001
QoR-15 day 7 (0–150)	128.3 ± 12.3	118.8 ± 13.8	<0.001
Postoperative nausea/vomiting	11 (17.7)	18 (29.5)	0.122
Postoperative ileus	7 (11.3)	22 (36.1)	0.002
Surgical site infection	7 (11.3)	7 (11.5)	0.973
Anastomotic leak	2 (3.2)	3 (4.9)	0.678
Any complication (Clavien–Dindo ≥ II)	12 (19.4)	27 (44.3)	0.003
Major complication (Clavien–Dindo ≥ III)	2 (3.2)	8 (13.1)	0.054
30-day readmission	3 (4.8)	5 (8.2)	0.486

**Table 4:** Subgroup outcomes by procedure type (ERAS vs. standard care).

Procedure Type	*n* (ERAS/Standard)	Ileus ERAS *n* (%)	Ileus Standard *n* (%)	*p*	LOS ERAS	LOS Standard	*p*	MME48 ERAS	MME48 Standard	*p*
Right colectomy	24/15	3 (12.5)	5 (33.3)	0.200	3.9 [3.4–4.9]	5.4 [4.7–6.9]	0.011	37.2 [25.1–45.4]	63.7 [40.5–85.5]	0.002
Left/sigmoid colectomy	25/31	2 (8.0)	12 (38.7)	0.014	4.4 [3.7–5.2]	5.7 [4.5–6.8]	0.002	31.5 [24.2–46.4]	60.6 [47.5–98.8]	<0.001
Anterior resection	13/15	2 (15.4)	5 (33.3)	0.386	4.5 [3.6–5.7]	5.5 [4.2–6.1]	0.211	41.8 [29.0–70.2]	66.3 [35.4–85.2]	0.133

Interaction *p*-values: POI interaction *p* = 0.379 (likelihood-ratio test); LOS interaction *p* = 0.609 (nested model comparison on log(LOS)).

**Table 5:** Spearman correlation matrix (ρ) for key process and recovery variables.

	ERAS Score	Opioids (MME48)	Time to Flatus	Length of Stay	QoR-15 POD2	Charlson Index	Op Time
ERAS score	—	−0.5 ***	−0.4 ***	−0.5 ***	0.3 ***	−0.1	−0.1
Opioids (MME48)		—	0.2 *	0.4 ***	−0.2 *	0.0	0.1
Time to flatus			—	0.4 ***	−0.2 *	0.1	0.1
Length of stay				—	−0.3 ***	0.2 *	0.2 *
QoR-15 POD2					—	−0.1	−0.2 *
Charlson index						—	0.1
Op time							—

(Blank cells indicate the lower triangle; — indicates the diagonal. * *p* < 0.05; *** *p* < 0.001.).

**Table 6:** Multivariable logistic regression for postoperative ileus.

Predictor	Adjusted OR	95% CI	*p*-Value
ERAS pathway (vs. standard)	0.2	0.1–0.7	0.013
Age (per 10 years)	1.2	0.8–1.7	0.425
ASA III (vs. I–II)	0.8	0.3–1.9	0.565
Operative time (per 30 min)	0.9	0.6–1.5	0.778
Conversion to open	0.3	<0.1–2.9	0.294
Opioids 0–48 h (per 25 MME)	1.7	1.2–2.5	0.006

Model AUC = 0.8.

**Table 7:** Exploratory adherence–outcome gradient across the cohort.

Adherence Category	*n*	POI, *n* (%)	LOS, Days	Opioids 0–48 h, MME	QoR-15 POD2
Score 2–4	27	11 (40.7)	5.8 [4.8–6.9]	71.2 [56.4–93.1]	100.6 ± 14.8
Score 5–6	33	10 (30.3)	5.2 [4.4–6.1]	57.9 [45.1–76.8]	105.2 ± 15.1
Score 7–8	35	6 (17.1)	4.5 [3.7–5.3]	40.6 [29.8–55.2]	112.8 ± 13.9
Score 9–10	28	2 (7.1)	3.9 [3.4–4.8]	28.7 [22.4–37.1]	119.6 ± 12.7
*p* for trend		<0.001	<0.001	<0.001	<0.001

## Data Availability

The data presented in this study are available on request from the corresponding author.
